# *Palaeoloxodon* and Human Interaction: Depositional Setting, Chronology and Archaeology at the Middle Pleistocene Ficoncella Site (Tarquinia, Italy)

**DOI:** 10.1371/journal.pone.0124498

**Published:** 2015-04-21

**Authors:** Daniele Aureli, Antonio Contardi, Biagio Giaccio, Brian Jicha, Cristina Lemorini, Sergio Madonna, Donatella Magri, Federica Marano, Salvatore Milli, Valerio Modesti, Maria Rita Palombo, Roxane Rocca

**Affiliations:** 1 Dipartimento di Scienze Fisiche, della Terra e dell'Ambiente, U.R. Preistoria e Antropologia, Università di Siena, Via Laterina 8, 53100, Siena, Italy; 2 Museo Civico A. Klitsche De la Grange, Palazzo Camerale, Piazza della Repubblica, 29, 00051, Allumiere, Roma, Italy; 3 Istituto di Geologia Ambientale e Geoingegneria, CNR, Via Salaria km 29,300, 00016, Monterotondo Stazione, Roma, Italy; 4 Department of Geoscience, University of Wisconsin-Madison, Madison, WI, United States of America; 5 Dipartimento di Scienze dell’Antichità, SAPIENZA Università di Roma, Piazzale Aldo Moro 5, 00185, Roma, Italy; 6 Dipartimento di Scienze e Tecnologie per l’Agricoltura, le Foreste, la Natura e l’Energia, Università di Viterbo, Via S. Camillo de Lellis, 01100, Viterbo, Italy; 7 Dipartimento di Biologia Ambientale, SAPIENZA Università di Roma, Piazzale Aldo Moro 5, 00185, Roma, Italy; 8 Dipartimento di Scienze della Terra, SAPIENZA Università di Roma, Piazzale Aldo Moro 5, 00185, Roma, Italy; 9 UMR 7041- ArScAn équipe AnTET, 21 allée de l’Université F-92023, Nanterre, Paris, France; Universidade do Algarve, PORTUGAL

## Abstract

The Ficoncella site in northern Latium (Italy) represents a unique opportunity to investigate the modalities of a short occupation in an alluvial setting during the Lower Palaeolithic. The small excavation area yielded a lithic assemblage, a carcass of *Palaeoloxodon antiquus*, and some other faunal remains. The main objectives of the study are to better characterize the depositional context where the *Palaeoloxodon *and the lithic assemblage occur, and to evaluate with greater precision the occupation dynamics. A 25 m-long well was drilled just above the top of the terrace of the Ficoncella site and faunal and lithic remains were analyzed with current and innovative techniques. The archaeological site contains floodplain deposits as it is located next to a small incised valley that feeds into a larger valley of the Mignone River. A tephra layer capping the site is ^40^Ar/^39^Ar dated to 441± 8 ka. Collectively, the geochronologic, tephrochronologic and geologic data, suggest the site was occupied during MIS 13. The new results should prompt further research at Ficoncella in order to improve our understanding of the dynamics of human settlement in Europe during the Early to Middle Pleistocene.

## Introduction

The human-elephant interaction during Lower Palaeolithic is an intriguing issue that has been the subject of a number of studies [1]. Various sources of evidence point out the contemporaneous presence of humans and proboscideans (and sometimes the exploitation of elephant carcasses) in Early and early Middle Pleistocene sites in Africa (FLK North Olduvai Bed I and II [2], Barogali [3], Olorgesailie [4], Nadung’a 4 [5], Mwaganda’s Village [6], Haïdalo [7], Kaddanarti [8], HP766 in Wadi Umm Rahau [9], El-Kherba, [10]) and in the Levant (Gesher Benot Ya’akov [11], Revadim Quarry [12–13]). In Southern Europe, the Early Pleistocene site of Fuente Nueva 3 (Guadix-Baza Basin, Orce, Spain), where a number lithic implements surround a *Mammuthus meridionalis* carcass, provides the oldest evidence of human-elephant interaction [14]. A southern mammoth is found at the Barranc de la Boella site (la Canonja, Spain), which has a lithic assemblage dated to about 1 million years ago [15]. The exploitation of *Palaeoloxodon* carcasses is documented in a number of Middle Pleistocene sites in Spain (Aridos 2, MIS 11 [16], Ambrona MIS 12 [17]) Italy (Notarchirico, early Middle Pleistocene [18], Castel di Guido, late Middle Pleistocene [19–20] and La Polledrara di Cecanibbio, [21]) and France (Terra Amata, [22]).

The transition from the Early to Middle Pleistocene represents a key period, marked by profound changes between the first appearance of human groups in South Europe [23–24–25–26–27–28–29–30] and the early use of handaxes [15–31–32–33–34]. In the earliest Acheulean assemblages of Italy, dated at about 600 ka (Notarchirico [32]), large cutting tools are poorly or not represented, whereas handaxes made of elephant bones were found at Fontana Ranuccio, a site dated at 450 ka [35]. The site of Ficoncella, in northern Latium, subject of the present study, is chronologically constrained to be between these two sites. The Ficoncella archeological layer, which yields a small lithic assemblage and a few mammal bones, including scanty remains of a *Palaeoloxodon antiquus* carcass [36], provides evidence of a short human occupation in an alluvial setting, possibly related to the presence of a dead elephant. New sedimentological and tephrochronologic data are used to reconstruct the depositional setting and constrain the age of the fossiliferous/archeological layer. In addition, the paper aims to better characterize the depositional environment where *Palaeoloxodon* and lithic implements occur, describe the technical characteristics and the spatial distribution of the lithic assemblage, and understand human-elephant interactions at the Ficoncella site.

## Geological Setting

### Regional geology

**Fig 1:**
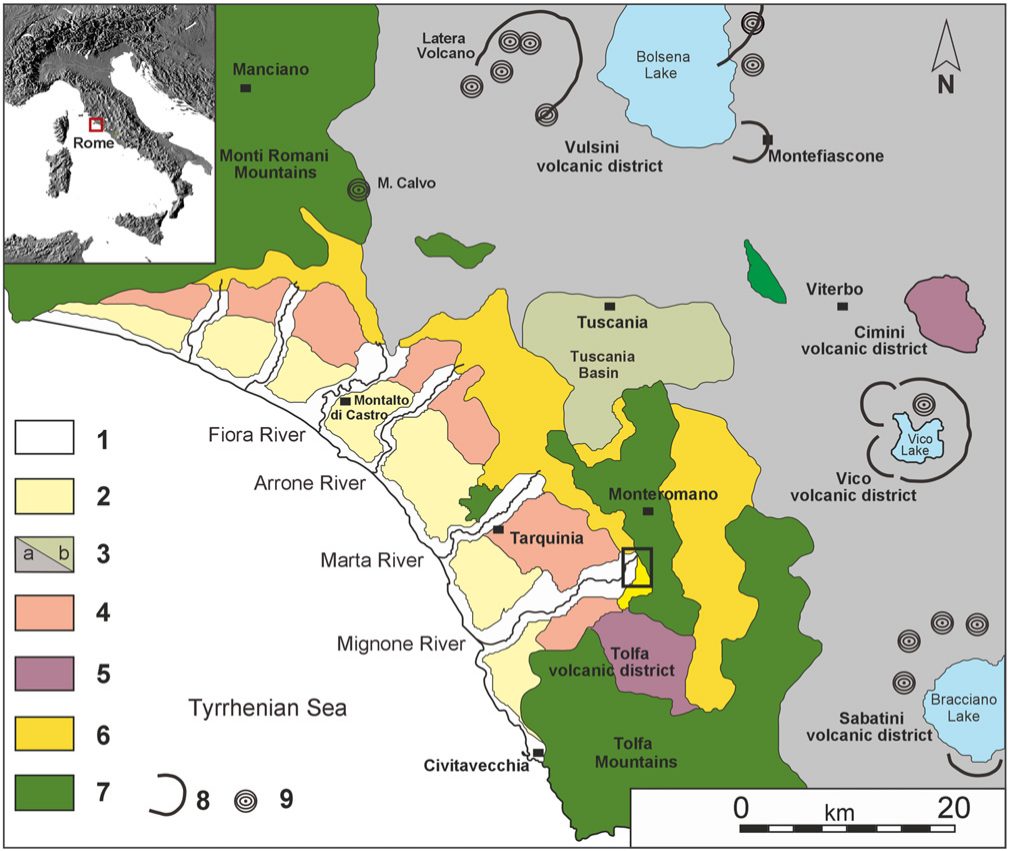
Geological sketch map of the northern Latium and location of the study area. 1) Holocene sediments; 2) Middle-Upper Pleistocene terraced marine sediments rich in volcanic components; 3a) Middle-Upper Pleistocene alkali-potassic lavas and volcaniclastic deposits; 3b) Middle-Upper Pleistocene volcaniclastic sediments of the Tuscania basin; 4) Lower Pleistocene marine sediments; 5) Acid volcanic rocks of the Tolfa and Cimini volcanic district; 6) Pliocene marine sediments; 7) Meso-Cenozoic flysch units of the Monti Romani and Monti della Tolfa; 8) Margins of the main craters and calderas; 9) Main scoria cones and small central edifices. (Modified from [55]).

The deposits at the La Ficoncella site are part of the Plio-Pleistocene Tarquinia basin [37–38–39–40–41], a NW-SE oriented basin, about 27 km wide, which extends for almost 40 km along the Latium Tyrrhenian margin (Fig 1).

The substrate of this basin is extensively deformed and is constituted of a series of stacked tectonic units related to the main orogenic phases of the Apennine chain. Following these phases, Middle-Upper Miocene extensional tectonics developed due to the opening of the Tyrrhenian back-arc basin [42–43–44–45]. This extensional phase caused the formation of a series of half-graben basins, mainly oriented NW-SE and subordinately NE-SW, which were filled with syn- and post-rift clastic sediments of Plio-Pleistocene age [46–47–38] with associated volcanic units belonging to the Roman Magmatic Province [48–49–50–51–52]. In the study area, the older volcanic activity (about 2 Ma) is sourced from the acid Tolfa and Cimini volcanic district (Fig 1). After a quiescence period, renewed ultra-potassic volcanic activity developed from 0.80 Ma to less than 0.04 Ma [53]. The products of this mainly explosive volcanic activity covered most the study area, reaching the coast and interacting with the coastal sedimentation through the Middle-Late Pleistocene [54–55].

All of the Tyrrhenian margin of Latium has been subject to general tectonic uplift since the Pliocene [56–57]. These movements were superimposed onto glacio-eustatic sea-level changes that produced a stack of sedimentary units that are progressively younger moving seaward [53–55]. From the geomorphological point of view, in the studied area, this uplift promoted the formation of several marine terraces [37–58–59–60–61–55]. In particular during the Middle-Late Pleistocene three marine terraces were recognized [55], whose deposits are rich in volcanic components deriving from the volcanoes of the Roman Magmatic Province. The deposits of these terraces, placed at elevations of about 20–30, 40–47, and 50–90 m a.s.l. were dated between 125 ka and 450 ka, allowing correlation with MIS 5, 7 and 9 respectively.

### Stratigraphical and depositional setting at the Ficoncella site

**Fig 2:**
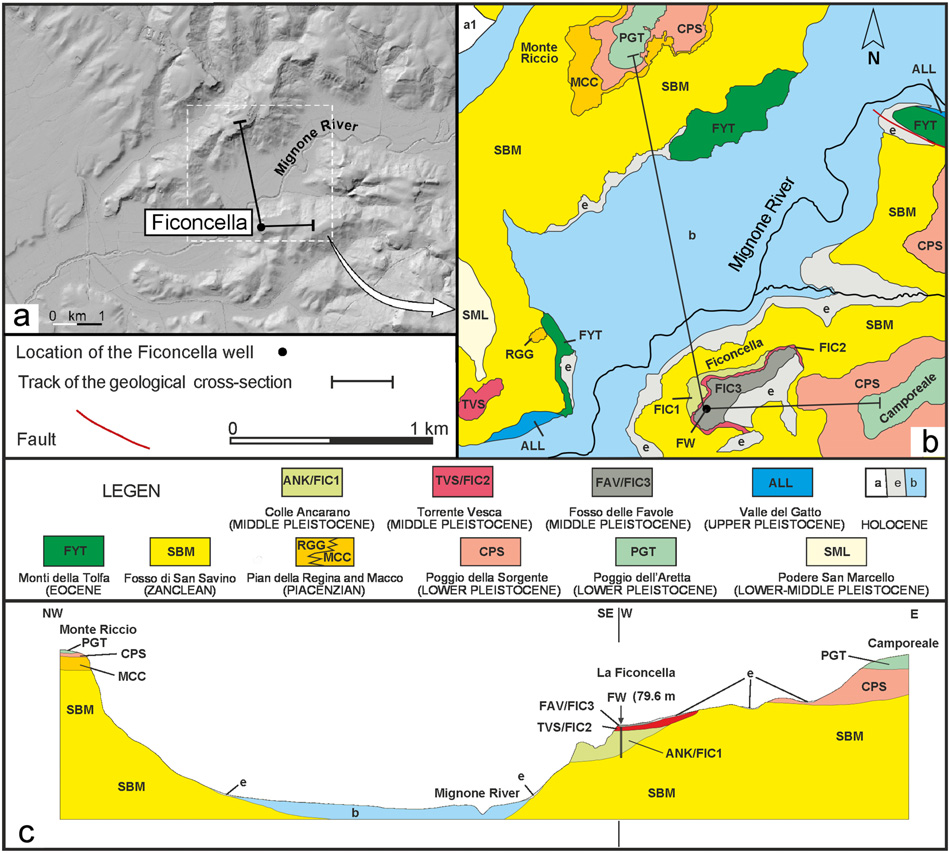
Digital elevation model of the Ficoncella site and geological sketch map of the study area. a) Digital elevation model of the Ficoncella site area showing the flat terraced morphologies characterinzing the northern sector of the Latium coast; b) Detailed geological map of the study area and c) geological cross-section. The codes are those reported in the official geologic map of the Italian Geological Survey (sheet 354 Tarquinia). A modification occured in the sector of the Ficoncella site where the new data reported in this work have allowed us to better differentiate the stratigraphic units. On this basis the FIC1, FIC2, FIC3 units have been tentatively correlated to the units occurring in the official geological map. Legend: FYT – sandstone and mudstone turbidites; SBM – marine clay and silty clay with local intercalations of conglomerates; RGG/MCC – clayey sands and bioclastic calcarenites; CPS – bioclastic sands; PGT – fluvial gravel with carbonate and volcaniclastic clasts; SML – calcarenites and bioclastic sands; ANK/FIC1 – fluvial sands and silt rich in volcaniclastic sediments; TVS/FIC2 – Ignimbrite deposits; FAV/FIC3 – fluvio-lacustrine sandy silt deposits; ALL – fluvial sands and gravel rich in volcanic clasts; a1 – landslide deposits; e – eluvial and colluvial deposits; b – alluvial deposits.

In a recent paper, Aureli et al. [36] investigated the uppermost part (ca. 10 m) of the stratigraphic succession cropping out along the left side of the Mignone River at the Ficoncella site (Fig 2), which form a terrace between 75 and 95 m a.s.l.

They distinguished three main depositional units. From lowest to highest they are named: FIC 1, FIC 2, FIC 3. The lowest unit (FIC 1), which contains the faunal remains, is 5 m thick and the base is unexposed. It is made up of sandy to silty sediments and an abundant volcaniclastic components including weathered ash, lapilli and volcanic minerals and in particular leucite crystals. Aureli et al. [36] attributed this unit to a generic fluvial environment. The intermediate unit (FIC 2) consists of a 2–3 m thick ignimbritic deposit. The lithologic features of this unit and the chemical analysis of the included glass scoria suggest that it is correlative to the “Tufo Rosso a Scorie Nere” pyroclastic flow deposits from one of the major eruptions of the Sabatini Volcanic Complex, dated 433 ± 6 ka by Cioni et al. [49] and 449 ± 1 ka by Karner et al. [52]. The uppermost unit (FIC 3) is about 2–3 m thick and is constituted by silty deposits with scattered, sub-rounded siliceous clasts.

Based on these data, Aureli et al. [36] suggested an age for the lower unit FIC 1 between the beginning of the ultrapotassic volcanic activity of the area (ca 800 ka: [52–53]) and ca 450 ka, although they considered the FIC1 fluvial fossiliferous deposits to be probably much closer to its upper temporal limit (ca 450 ka) than to its lower one (ca 800 ka). A refinement of this age was based on the correlation of the top of the FIC 3 unit with the remnants of a terraced surface on the adjacent right side of the Mignone River, placed between 80 and 100 m a.s.l., at an elevation comparable to the Ficoncella site. The deposits constituting this terrace were attributed to the synthem 4 of De Rita et al. [55], which contains, in its uppermost portion a Plinian fall deposit attributed to the “Vico α” eruption from the Vico volcano, dated between 420 and 400 ka [49]. On the basis of these considerations Aureli et al. [36] suggested an age between 500 and 400 ka for the whole sedimentary succession cropping out at Ficoncella thereby covering the interval from MIS 13 to MIS 11.

## Material and Methods

### Ethics Statement

All necessary permits were obtained from the Italian Ministery of Culture (Soprintendenza per i beni archeologici dell’Etruria meridionale, P.le di Villa Giulia 9, 00196 Roma), for the excavation (field permit number: MA1037-MA1446), and for the study of lithic and faunal implements, which complied with all relevant regulations (lithic specimen number: 409; fauna specimen number: 42). The specimens are temporary housed at the Museo Civico De La Grange di Allumiere (Rome), piazza della Repubblica, 29, Allumiere, Rome Italy.

### Stratigraphic and sedimentological analysis

**Fig 3:**
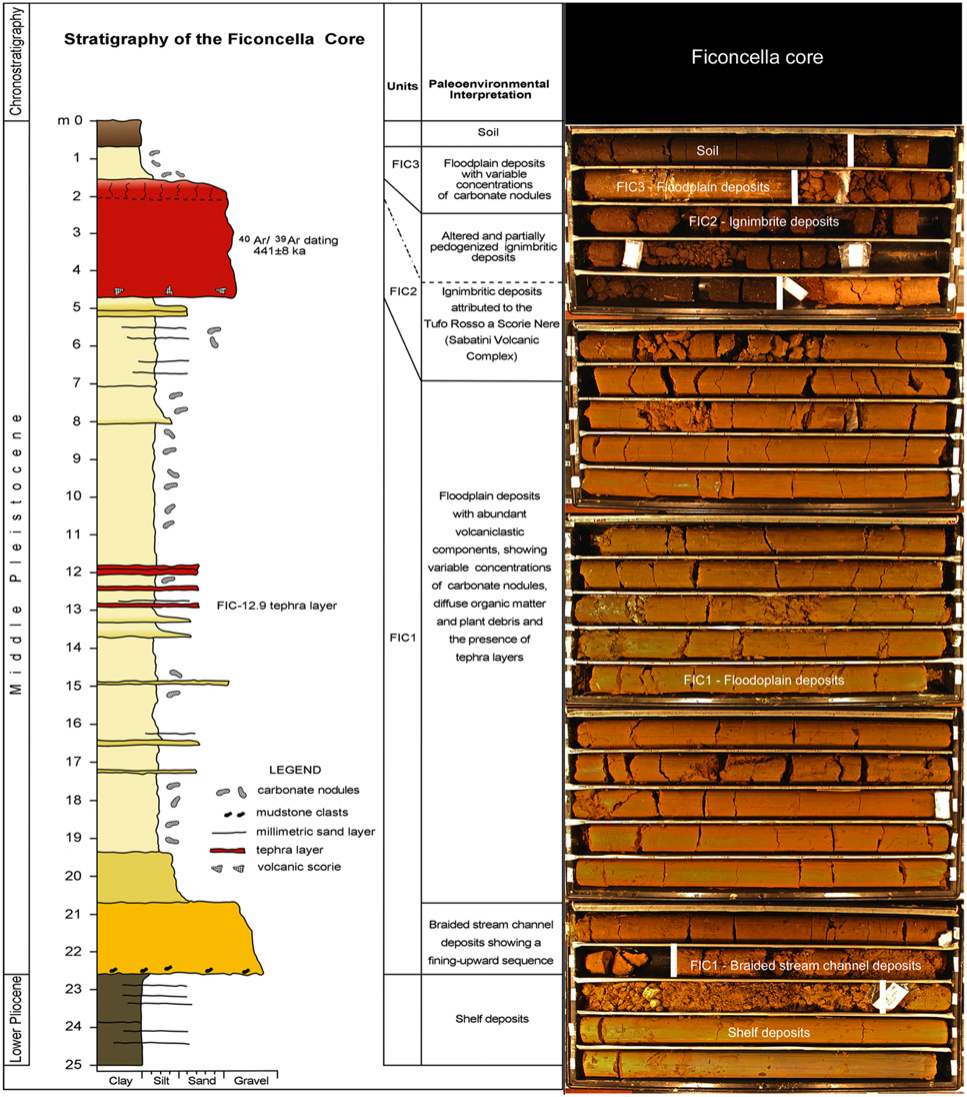
Stratigraphic column of the Ficoncella core showing the inferred depositional environment and pictures of the core.

With the aim of refining the stratigraphy and the sedimentological characteristics of the Ficoncella site, a 25 m-long well was drilled into the terrace above the site (Fig 3).

The drilling method consisted of a double corer, having a diameter of 12 cm; the recovery of the core was more than 95%. The description of the core was made after it was split into two parts for its entire length. Grain size and other textural attributes, as well as sedimentary structure and colour were described. Other aspects such as the presence of shelly debris, clasts of clay, organic matter, wood fragments, and roots were also taken into consideration as useful features for the paleoenvironmental interpretation. Pollen analysis was carried out on hyena coprolites, but, unfortunately, it did not reveal any appreciable data; further palynological investigations are in progress.

### ^40^Ar/^39^Ar dating

^40^Ar/^39^Ar dating was performed on sanidine grains extracted from the outcropping ignimbrite unit (FIC 2 unit). Sanidine phenocrysts (~ 150 μm) were isolated from the ignimbrite using standard magnetic and density separation techniques, and were co-irradiated with the Fish Canyon Tuff sanidine standard (age, 28,201 Ma: [62]) at the Oregon State University TRIGA reactor in the Cadmium-Lined In-Core Irradiation Tube. At the University of Wisconsin-Madison Rare Gas Geochronology Laboratory, sanidine samples and standards were fused using a 25W CO_2_ laser, following the methods of [63]. Mass discrimination was assessed by analysis of air pipette aliquots throughout the analytical session, and was calculated relative to a ^40^Ar/^36^Ar ratio of 295.5 [64].

### Electron microprobe analysis

Major and minor element compositions were determined on micro-pumice fragments and/or glass shards (grain size >250 μm) from the tephra layer FIC-12.9 (Fig 3). The analyses were carried out at the Istituto di Geologia Ambientale e Geoingegneria of the Italian National Research Council (IGAG-CNR) (Rome, Italy) using a Cameca SX50 electron microprobe equipped with a five-wavelength dispersive spectrometer. Operating conditions were as follows: accelerating voltage, 15 kV; beam current, 15 nA; beam diameter, 10–15 μm; counting time, 20 s per element. The following standards were used: wollastonite (Si and Ca), corundum (Al), diopside (Mg), andradite (Fe), rutile (Ti), orthoclase (K), jadeite (Na), phlogopite (F), potassium chloride (Cl), baritina (S), and metals (Mn). The Ti content was corrected for the overlap of the Ti-Kα peaks. To evaluate the accuracy of the EMP analyses, three international secondary standards (Kakanui augite, Iceladic Bir-1, and rhyolite RLS132 glasses) were analysed prior to the measurements. The mean analytical precision was <1% for SiO_2_ and Al_2_O_3_, 5% for K_2_O, CaO and FeO, and 6–9% for the other elements.

### Archaeological excavation

We led a planimetric excavation, which was very slow because of the presence of very small lithic flakes. First, it was necessary to divide the excavation surface in two areas (A and B), which correspond to different dispersions of lithic and faunal material. Area A contained an elephant skull and a concentration of the small lithic artefacts; area B contained a concentration of the other bones of elephant (vertebra, pelvis), and lower concentration of lithic artefacts. To be able to record all the stratigraphic evidence and the distribution of the material concentrations, we decided to lead the excavation by artificial level (5 cm). Thanks to this very precise procedure even the small pieces were positioned in the 3D maps. For taphonomical reasons and to approach the spatial repartition of the material, all the finds were coordinated (x, y, z). We also indicate for each piece its inclination (north, south, east, west, vertical, horizontal) and the direction (north-south, east-west, north-east/south-west, north-west/south-east). Due to the sedimentary context and the characteristic of the lithic industry, all the sediments were sieved (1mm). This was very important to understand the lithic assemblage and the human activities held on the site (retouch flakes, small flake for core preparation).

### Lithic industry analysis

In order to evaluate the human behaviour on the site, the lithic assemblage was analysed by means of a classical technological approach [65–66]. This classical techno-production analysis was combined with a techno-functional approach [67–68–69]. The goal of this method is to understand the functional potential of a tool thanks to the chronology of the removals, and the technical consequences of each removal on the blank (angles, surfaces morphology, etc.). Refitting analysis and a RMU (Raw Material Unit) approach [70] are important steps in understanding the lithic industry and the spatial distribution of the material. This approach was also combined with a use wear analysis (for a recent overview of the method see [71]) allowing a better understanding of the site function and interactions with the fauna.

## Results

### Stratigraphy and sedimentary facies description of the Ficoncella core

**Fig 4:**
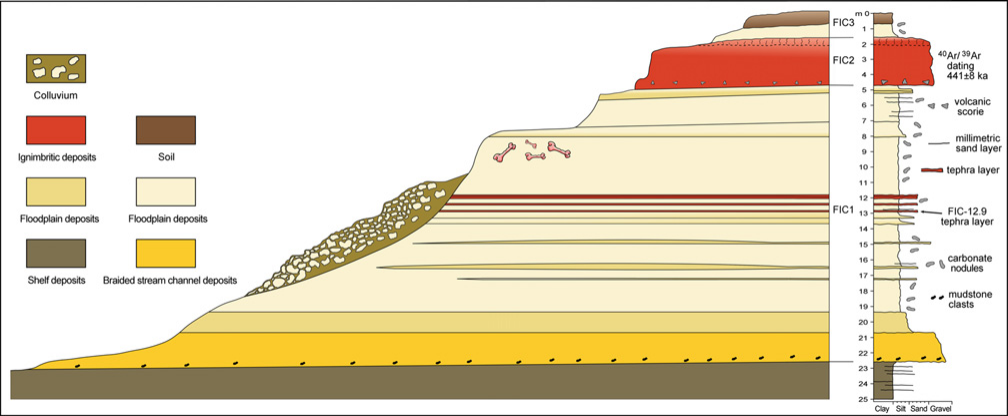
Stratigraphic scheme of the Ficoncella site.

The Ficoncella well (Figs 3 and 4) crosses a succession including, from bottom to top, Lower Pliocene marine sediments forming the substrate on which the terraced deposits of the Ficoncella site lie, and a complete record of deposits forming the three previously recognized units FIC1, FIC2, and FIC3.

These units are separated by two important erosional surfaces: 1) the first surface marks a significant hiatus, evidenced by a sharp change of facies between the Lower Pliocene grey mudstone deposits and the FIC1 deposits (continental in origin); 2) the second surface is placed at the base of the FIC2 unit. Two main facies associations attributed to channelized deposits and to floodplain deposits have been recognized. The channel deposits lie above the first erosional surface, between 22.60 m and 20.35 m depth (Fig 3), and are characterized by two superimposed coarse-grained facies. The lower facies is constituted by sandy-gravel deposits with intraformational mud clasts derived by the erosion of the underlying unit (marine Lower Pliocene deposits). The upper facies is constituted by very coarse sand with gravel lenses; the passage between these facies is transitional. Gravel clasts are subrounded to rounded, having variable shape from moderately elongate to moderately flat (terminology from Blott and Pye [72]). These deposits are moderately to poorly sorted and contain a reduced quantity of matrix, so that the resulting framework is clast-supported. The composition of gravel clasts suggests they come from different sources. These coarse facies sharply grade upward (between 20.69 m and 20.35 m depth) to a clayey silt with local presence of thin layers of very fine sand. No evidence of tractive structures was recognized in these deposits.

Based on the textural features of these deposits we interpret these sediments as the product of deposition in a shallow isolated to slightly braided stream channel, where stream-energy is commonly high and discharges episodically [73]. This is reflected in the lack of a well developed stratification and other cross-bedded characters within the deposits, although this facies sequence typically displays a fining-upward trend. The sharp passage from the coarse to fine deposits suggests a rapid abandonment of the channel, probably related to lateral migration, a process that could be connected to avulsion from different mechanisms: flood event, base level change, or climate change.

The floodplain deposits constitute the main portion of the core, extending between 0.70 and 20.69 m, with the only exception being between 1.55 and 4.70 m where the ignimbrite unit (FIC 2 of Aureli et al. [36]) occurs (Fig 3). The floodplain deposits are made up of silty clays and clayey silts with diffuse fine and very fine sands, often forming thin layers with a sharp base. The latter together with the clayey silt often form centimetre scale fining-upward sequences. Organic matter is locally widespread in the sediment or concentrated in some layers. Carbonate nodules of irregular or nearly spherical shape are more or less widespread throughout the core although they show a higher concentration between 7 and 13 m. The volcanic material is an important constituent of the entire core although individual tephra layers have been recognized between the following depth intervals: 11.90–12.05 m, 12.80–12.90 m, 14.90–14.95 m and 16.30–16.35 m. All these layers consist of well-sorted, clast-supported ash- to lapilli-sized deposits with a sharp basal contact onto the underling fluvial sediment that indicate a primary fallout deposition. The lapilli-ash pumices composing these fall deposits are often strongly altered, with exception of the tephra at 12.80–12.90 m-depth, which is constituted by glassy ash and millimetric pumices suitable for tephrostratigraphic analyses (see the details below).

All these features suggest a floodplain environment, in which sedimentation was strictly related to flood events alternating with drying and subaerial exposure, depending on climate, water table and vegetation [74–75]. In particular, we interpreted the small fining-upward sequence as a product of decelerating flows, related to overbank processes, which deposited sediments with diminishing grain size distally from the channel. The general light brown colour of the sediments suggests an oxidized environment, also highlighted by the diffuse and low concentration of organic matter that might indicate less prolific vegetation. These characters highlight an incipient pedogenesis of these deposits, a process rather common in an actively aggrading floodplain environment, strictly related to the seasonal migration of the water table. These type of soils show some disturbance by roots and, in some cases animal bioturbation on the temporarily exposed surface.

One of the more interesting features in the floodplain deposits is the presence of calcareous nodules that, although present at different depths in the core, are especially concentrated between 7 and 11 m. This interval is where mammal and archaeological remains were found. Their presence highlights a migration of water from the upper to lower levels in deposits where precipitation of soluble minerals occurs [76]. The depth of the carbonate nodular horizon in soils has been correlated with the mean annual precipitation and seasonality [77–78–79–80–81]. In general, the horizon where calcareous nodules are developed show different thicknesses, being deeper in subhumid regions and shallower in semiarid regions. We utilized this feature to better characterize the climatic environment of the floodplain deposits of the Ficoncella core. Taking into account the different concentrations of calcareous nodules in the core, we hypothesize that the lower part of the core where nodules are sparse or rare was deposited during a period of semiarid conditions. In contrast, subhumid conditions are inferred for the upper part of the core. Just below the FIC 2 ignimbrite unit (between 7 and 11 m), where the highest concentration of calcareous nodules is recorded, is where palustrine conditions probably existed.

The above data and interpretations allow us to reliably outline the depositional context of the Ficoncella site as the filling of a small incised valley tributary of the larger incised valley of the Mignone River. Both valleys record the effects of the eustatic sea-level changes in the Quaternary and of local tectonic effects, producing a characteristic stacking pattern of the depositional sequences formed along the Latium continental margin [54–55–82–83] see geological setting.

The aggradation shown by the Ficoncella core is coherent with a rise of the base level that promoted the initial avulsion of the braided channel at the base of the valley, its upland migration and transformation into a sandy meandering channel with a well developed floodplain. The arrival of the ignimbrite unit probably covered the filling of the incised valley that was already partly eroded, as evidenced by the erosional surface that separates the FIC 1 and the FIC 2 units.

### Tephrochronology

#### Ignimbrite FIC 2

All eleven ^40^Ar/^39^Ar single crystal measurements of the sanidine grains from FIC 2 ignimbrite yield consistent dates sharing a weighted mean age of 441± 8 ka (Table 1).

**Table 1:** Major-element compositions (normalised to 100 wt%) of glass shards and/or micropumices from the investigated FIC-12.9 tephra.

SiO_2_	54.82	54.83	54.88	54.10	54.93	54.83	55.37	53.89	60.47	58.03	60.10
TiO_2_	0.61	0.61	0.64	0.63	0.59	0.66	0.59	0.65	0.40	0.50	0.55
Al_2_O_3_	19.16	19.36	19.43	19.28	19.34	19.49	19.54	20.62	19.07	19.06	19.46
FeO	5.03	5.24	4.79	5.05	4.69	5.14	4.49	4.82	2.78	3.91	3.08
MnO	0.23	0.24	0.19	0.22	0.16	0.13	0.21	0.23	0.24	0.10	0.10
MgO	1.44	1.48	1.44	1.50	1.30	1.39	1.22	1.41	0.38	0.80	0.38
CaO	5.66	5.73	5.84	5.81	5.67	5.83	5.15	5.72	2.69	4.09	2.99
Na_2_O	4.37	4.62	4.59	3.85	4.69	4.62	4.98	4.69	3.44	3.21	3.83
K_2_O	8.40	7.65	7.98	9.32	8.40	7.71	8.19	7.71	10.51	10.14	9.48
P_2_O_5_	0.30	0.24	0.23	0.24	0.22	0.21	0.25	0.26	0.03	0.17	0.04
F	0.52	0.55	0.35	0.40	0.50	0.35	0.37	0.37	0.35	0.13	0.36
Cl	0.10	0.12	0.13	0.09	0.10	0.09	0.14	0.10	0.11	0.08	0.14
SO_3_	0.32	0.39	0.32	0.29	0.45	0.30	0.52	0.26	0.09	0.18	0.14
Analytic Tot.	97.98	96.23	98.36	98.63	97.53	98.07	96.56	94.29	98.36	93.76	94.34
SiO_2_	54.72	57.53	55.01	54.89	55.01	55.61	54.73	54.53	53.82	54.58	54.85
TiO_2_	0.70	0.52	0.59	0.64	0.65	0.66	0.76	0.75	0.67	0.73	0.73
Al_2_O_3_	19.09	20.14	19.44	19.33	19.60	19.17	19.17	19.49	19.14	19.31	19.30
FeO	5.20	3.58	4.80	4.99	5.18	5.22	5.62	5.21	5.29	5.39	4.95
MnO	0.18	0.14	0.22	0.14	0.15	0.18	0.23	0.23	0.26	0.18	0.21
MgO	1.60	0.73	1.37	1.34	1.46	1.48	1.49	1.59	1.48	1.50	1.46
CaO	5.92	3.74	5.53	5.44	6.00	6.14	6.36	5.61	5.98	5.91	5.64
Na_2_O	4.68	3.44	4.38	4.74	3.76	4.80	5.13	4.83	4.76	4.42	4.57
K_2_O	7.59	10.02	8.44	8.26	7.97	6.46	6.21	7.43	8.31	7.75	8.03
P_2_O_5_	0.33	0.15	0.22	0.23	0.22	0.29	0.30	0.34	0.29	0.21	0.26
F	0.35	0.45	0.47	0.46	0.40	0.36	0.62	0.65	0.45	0.47	0.44
Cl	0.10	0.10	0.10	0.14	0.11	0.09	0.12	0.10	0.14	0.13	0.11
SO_3_	0.33	0.15	0.44	0.32	0.35	0.38	0.29	0.26	0.38	0.44	0.22
Analytic Tot.	94.50	93.84	96.21	97.33	96.81	97.04	98.30	98.93	92.99	95.94	97.17

The uncertainty on each single crystal fusion date is relatively high because the sanidine from this distal deposit is small (~100–150 microns), thereby containing very little Ar. The 441 ± 8 ka age is slightly younger than Tufo Rosso a Scorie Nere Sabatino (TRSN, 452 ± 1 ka [52] age recalculated relative to 28.201 Ma Fish Canyon standard), from the Mt. Sabatini volcanic district [84]. Although the ages of the two deposits are distinguishable, we support the previous attribution of the FIC 2 ignimbrite to the TRSN based on the glass major element composition [36].

### Layer FIC-12.9

**Fig 5:**
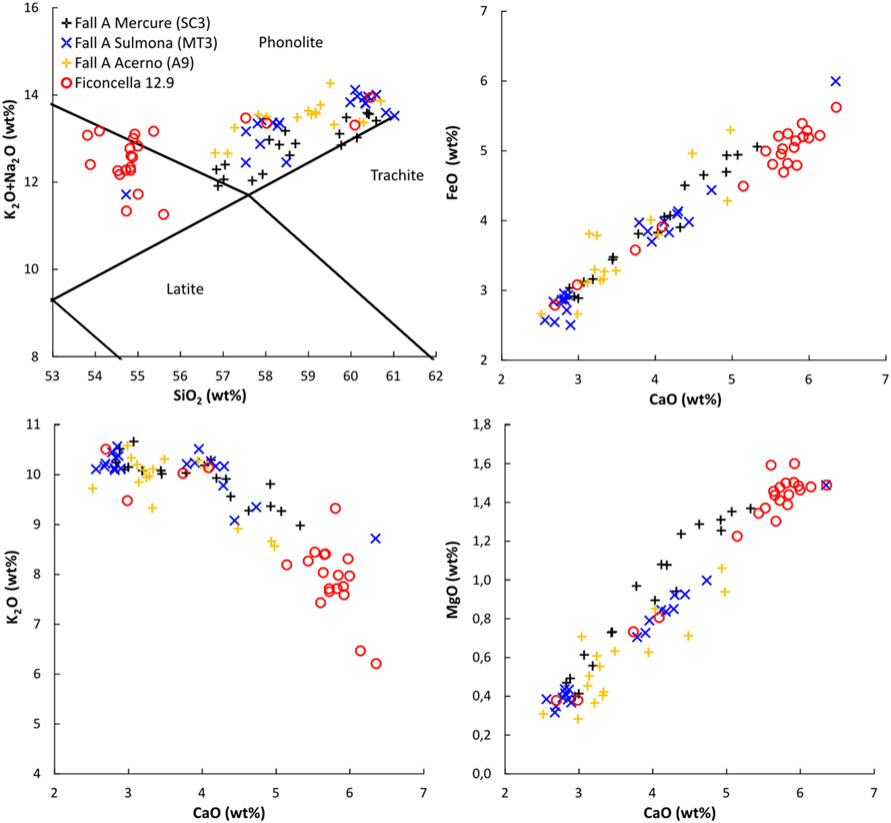
Compositional features of the investigated tephra (FIC-12.9) and of its proximal and distal counterparts. Total alkali *vs* silica and representative Harker diagrams (EMPA or EDS compositions) for glass from the tephra FIC-12.9 and from the distal equivalent tephras of the Mt. Sabatini Fall A pumice unit (~499 ka, [53]) from Mercure (layer SC3, [86]) and Sulmona basins (layer MT3, [114]) here also correlated to layer A9 from the Acerno basin [87].

This tephra, found at the depth between 12.90 m and 12.80 from the core top (Fig 3), is a 10 cm-thick fallout layer made of up to medium lapilli-sized (Ø 4–16 mm) vesicular, sub-aphyric, whitish pumices and poorly vesicular, leucite-bearing grey scoria. Sanidine, clinopyroxene, and biotite crystals and reddish thermally metamorphosed clay lithics complete the component assortment. Glass from this tephra is mainly tephri-phonolitic in composition, but overall has a relatively wide range in SiO_2_ content (~54 wt% to ~61 wt%; Table 1, Fig 5), ranging from phonolite to tephri-phonolite (Fig 5).

**Fig 6:**
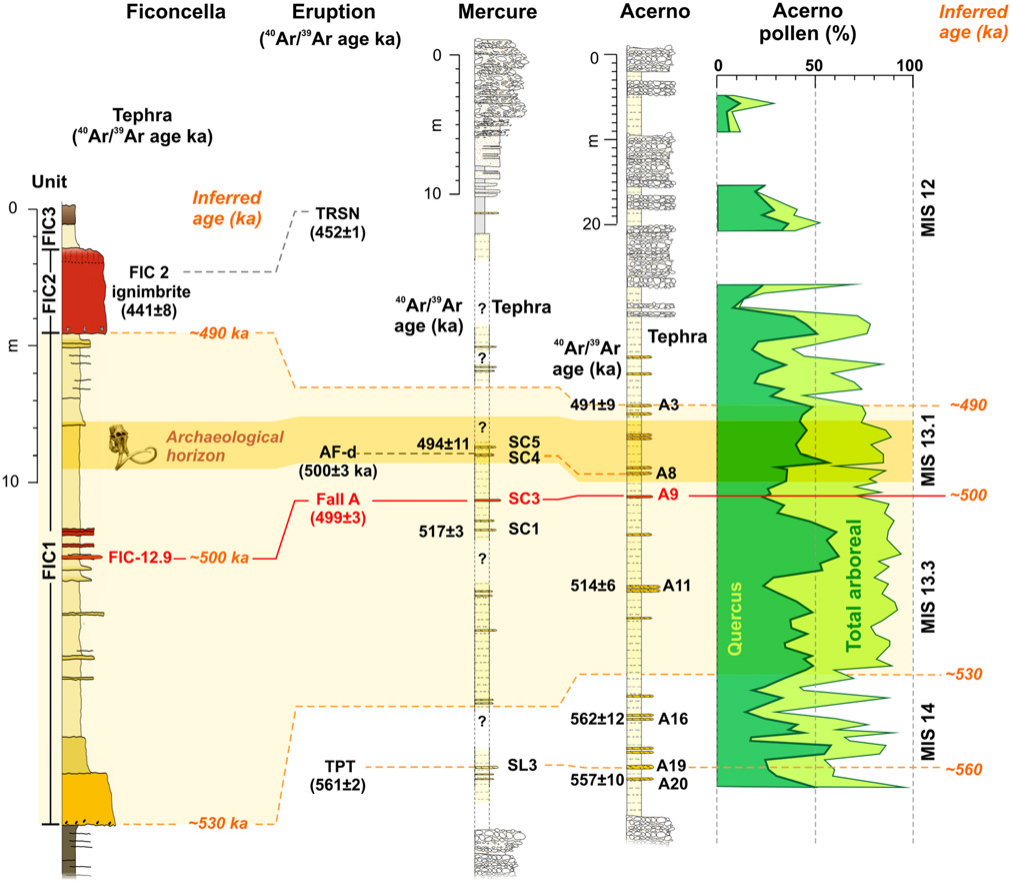
Climatostratigraphic position and age of the archaeological horizon inferred from the tephrostratigraphic correlation between Ficoncella and other successions containing the Sabatini Fall A pumice unit (~499 ka). Mercure tephra record and ^40^Ar/^39^Ar ages of the proximal correlated units (AF-d; Fall A and Tufo Pisolitico di Trigoria, TPT) from ref. [86], and reference therein; Acerno tephra-pollen record and ^40^Ar/^39^Ar tephra ages from refs. [87–88]. The possible succession of the marine isotope stages (MIS), from MIS 14 to the MIS 12, is also shown.

Major element composition of the glass from the FIC-12.9 tephra, its stratigraphic position ~4.70 m below the base of the FIC 2 ignimbrite – here dated to 441 ± 8 ka – as well as its textural and lithological features are consistent with the Fall A pumice unit, from a Plinian eruption in the Mt. Sabatini volcanic district [85]. This widespread tephra, dated to 499 ± 3 ka [53], was recently traced in distal settings of the Mercure and Sulmona Apennine intermountain basins [86] (Fig 5). Specifically, the occurrence of the idiosyncratic reddish lithics in the layer FIC-12.9, which is a diagnostic character for the Fall A proximal deposits [85], makes this correlation virtually unambiguous. The distal equivalent tephra of the Fall A unit from the Mercure basin (layer SC3, [86]), here also correlated to the layer A9 from the Acerno basin [87], is chronologically constrained by the consistent pair of ^40^Ar/^39^Ar ages of 516.5 ± 3.6 ka and 493.1 ± 10.9 ka (Mercure, [86]) and of 514.2 ± 5.6 ka and 491.3 ± 9 ka (Acerno, [87]), thus further strengthening the proposed correlation. In addition, according to the pollen record from the Acerno basin [88–87], the layer A9 (= FIC-12.9) was deposited during a stadial phase, which preceded a period of increasing frequencies of the *Quercus* pollen that may be correlated with the culmination MIS 13.1 climax dated at ~ 490 Ka [89] (Fig 6).

### Fauna

**Fig 7:**
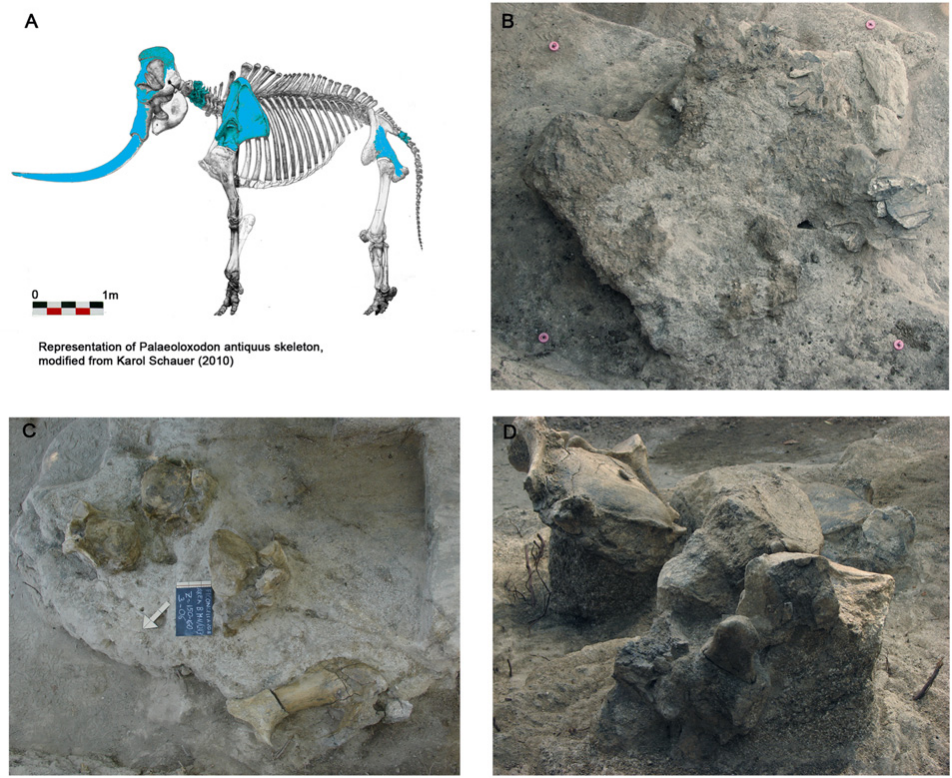
Faunal remains belonging to an individual of Palaeoloxodon discovered during the excavation companions. A: in blue are represented anatomical elements found until now; B: detail of the skull of Palaeoloxodon; C: excavation area south in which is has a concentration of several anatomical portions of Palaeoloxodon; D: Detail of two vertebrae in anatomical connection.

In the Ficoncella site, faunal remains consist of bones belonging to a single elephant carcass and few largely fragmented mammal bones. Elephant remains include a large part of a seriously damaged skull still partially buried in sediments (apex is not preserved, premaxillary bones are incomplete), a nearly complete left tusk, the proximal portion of the right tusk, the axis, two incomplete cervical vertebrae (C3 and C4) and two proximal caudal vertebrae (Fig 7).

The elephant can confidently be identified as *Palaeoloxodon* based on the morphology of the fan-shaped premaxillary bones and the pattern of Schreger lines (the visual artefacts that are evident in the cross-sections of tusks). The inner Schreger lines, faintly discernible around the pulp cavities, run slightly deviating from the ray of the section, while the outer Schreger lines, easily visible in the area closer to the cementum-dentine interface, markedly bent, strongly deviating from the ray. Consistently, the average amplitude of the concave (opening to the medial/inner area) and convex angles (opening to the lateral/outer area) ranges from about 60° (near the pulpal cavity) to 120° (outer Schreger angles closest to the cementum). This pattern is most frequently found in tusks of adult *Palaeoloxodon antiquus* [90]. Size and curvature of the tusks seem to be closer to the typology of tusks more frequently found in skulls showing a developed parieto-frontal crest, markedly overhanging the frontal bones (straight-tusked elephants of the so-called “*namadicus* morph” by [91]) than those, slender and gently curved, characterizing the skulls showing a less developed parieto-frontal crest (the so-called “Stuttgart morph” by [91]) (see [92–36] for a discussion).

The presence of tusks only slightly rotated in the alveoli with respect to their anatomical position, the moderate displacement of disarticulated bones, and a few gnawing marks visible on vertebrae and pelvic girdle indicate that the elephant carcass was possibly buried not long after death and did not suffer any transport by water. A large coprolite indicates the presence of a large carnivore (likely the spotted hyaena *Crocuta crocuta*).

A few remains of a large mammal are sparsely found in the sandy sediments of FIC 1 (*Equus* sp,? *Hippopotamus* sp., *Dama* sp. and *Bos primigenius*) and are fragmented and show taphonomic signatures suggesting either moderate weathering (e.g. fragments showing initial cracking parallel to bone fibre structures) or transportation (e.g. abraded bone splinters showing scratches by drag) or even a possible reworking from older sediments as confirmed by the presence of fossil balanid reworked from the lower Pleistocene marine sediments. However, a few small mammal remains (a nearly complete left humerus of *Talpa europea* and an upper incisor of a middle-sized rodent) are quite well preserved.

The taphonomical assessment of faunal remains is largely consistent with the palaeoenvironmental reconstruction inferred by geological and sedimentological data. The elephant carcass was possibly partially trapped in floodplain sediments. The exposed bones maybe suffered weathering alteration and carnivore attack. A few bones were moderately displaced or transported for a short distance. Other bones were transported and deposited by weak streams from the alluvial plane and/or from eroded older deposits.

### Lithic industry

The uncovered lithic assemblage consists of 409 pieces, including 296 small flakes and debris (< 5 mm) (Table 2).

**Table 2:** Technological composition of the assemblage.

Technological categories	Cores	Cortical flakes	Produced flakes	Shaped tools	Retouch flakes	Knapping debris	Total
	5	13	38	4	53	296	**409**

**Fig 8:**
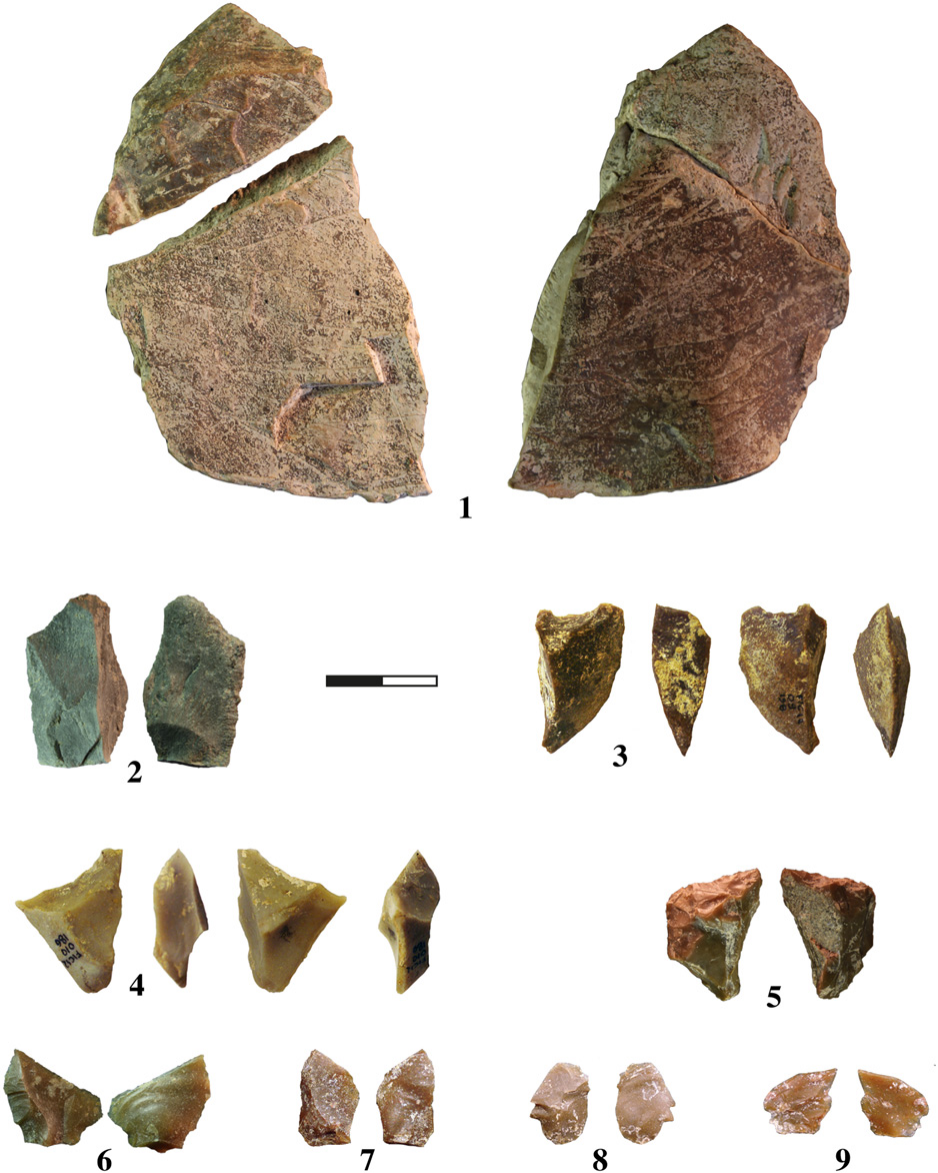
Photo of lithic artefact. 1 and 2: Limestone flake; 3 to 5: small flint tool; 6 to 8 small flint flakes; 9: retouch flint flake.

The implements were found in an area of about 6 m^2^, which is a small part of the entire site. Generally the state of preservation is pretty good, except for the limestone artefacts. Preliminary taphonomical analysis shows very few mechanical alterations, that is scars caused by trampling or by compression forces during deposition. The delineation of the tools is generally very well preserved thus making possible the determination of macro-traces of use, that is edge-removals and macro edge-rounding (Fig 8).

The first study of the composition of the assemblage shows the presence of different technological categories: shaped tools (no handaxes), cortical flakes, production flakes, retouch flakes, cores, and small debris (Table 2).

### Raw material

The presence of dorsal cortex on some implements, together with the presence of residual cores, attests to the procurement and exploitation of two different types of cobbles: small marine/fluvial pebbles and big cobbles of limestone (Table 3).

**Table 3:** Raw material repartition of the assemblage (excluding knapping debris).

Raw material	Flint	Limestone	Quartz	Total
	101	6	6	**113**

The limestone raw material seems to be local, as it is present today near the site. The small pebbles are principally flint and more rarely quartz. The precise provenance of those pebbles is unknown as this raw material is absent in this region during the remainder of the Palaeolithic. It may have been available during Lower Palaeolithic in floodplain or terraces. Thanks to several macroscopic parameters of the stones and the RMU (Raw Material Unit) approach, it was possible to identify twenty-one different cobbles imported to the site.

### Reduction sequences

**Fig 9:**
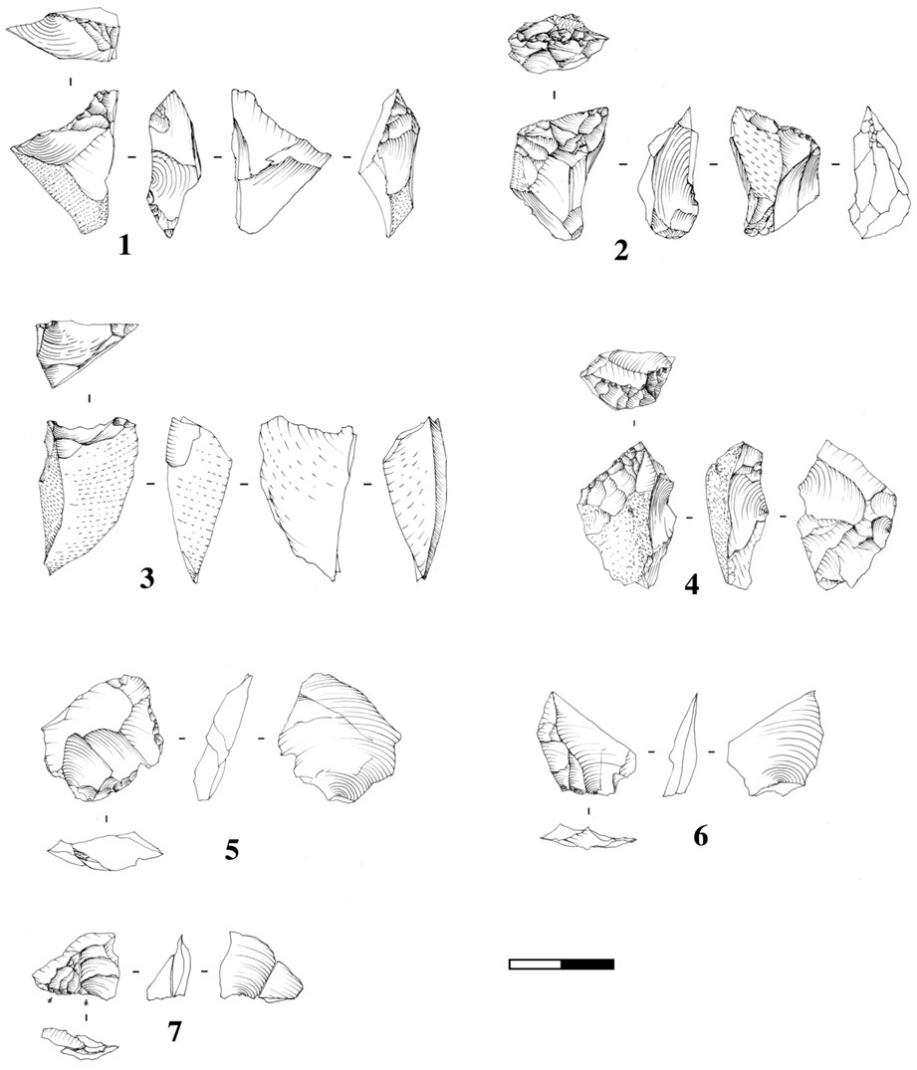
Examples of artefacts discovered during the excavation of Ficoncella site. 1 to 4: small shaped tools products from small flint pebbles; 5 and 6: unretouched flakes produced from small flint pebbles; 7: refitting of two small retouch flint flakes.

According to the technological and typometrical analysis, different reduction sequences are represented. Flint and quartz were preferred in order to obtain small flakes. The limestone has been exploited for the production of medium/large size flakes. Because there is only one core and the assemblage is only partially complete, we are not able to accurately reconstruct the knapping methods. The small shaped tools were obtained from the small pebbles. The presence of numerous flint retouch flakes, probably coming from a large tool, indicates a fourth reduction sequence (Fig 9).

In all the reduction sequences there are clearly some missing phases. It is not possible to now if the absence of phases is due to the partiality of the excavation or to selection of certain material by humans.

### Functional aspects

From a technological point of view, it is possible to identify four main goals that may have a functional potential. The first one includes large unretouched flakes on limestone. They offer a large length of cutting edge with a relatively sharp angle. Conversely, the very small shaped tools present a narrow cutting edge with a very open angle, a spine and a lateral cortical back. The presence of small flakes and retouched tools (as indirectly evidenced by the small retouch flakes) may offer some other functional possibility that is currently unrecognizable because of the incompleteness of the assemblage.

Thanks to the conservation of the lithic material, it was possible to use wear analysis for all the assemblage. The use wear analysis is still in progress, but the first results point out some edge-removal developed during use. These traces concern different technological categories and testify, in one case, to a working of material of medium hardness probably wood, and various actions of cutting on soft material probably on meat. The presence of small flakes in association with elephant remains is attested by use wear analysis during the Lower Palaeolithic in other sites, such as La Polledrara [93].

### Spatial distribution

**Fig 10:**
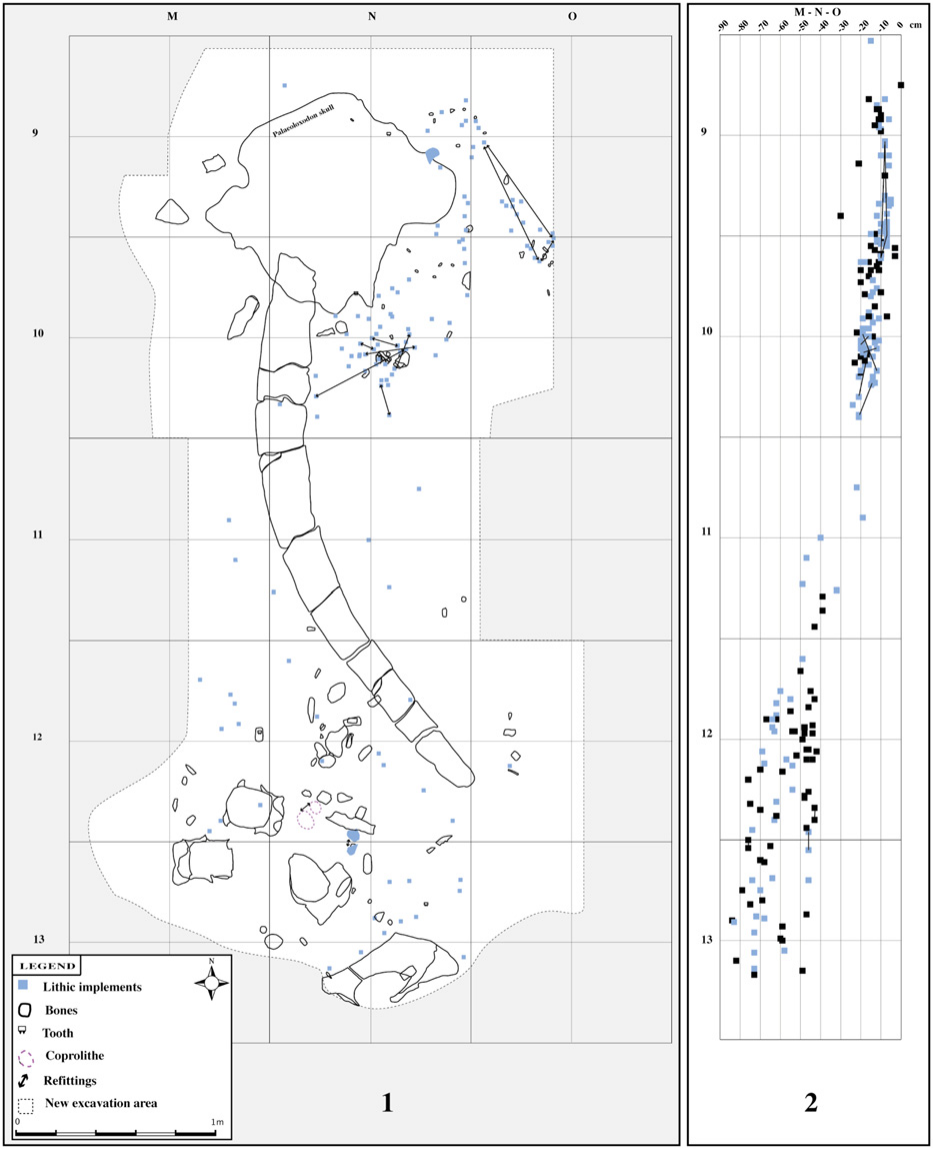
Plan of excavation with the artefacts found during the excavation campaigns 2010, 2011 and 2012. 1: Even if the excavation area is very partial, there are two major concentrations of artefacts: around the skull of Elephas and around the vertebrae of the same individual. 2: Vertical distribution of the archaeological remains. We can see that the lithic and faunal remains are at the same altitude.

The spatial distribution of the lithic elements is very instructive. The great majority of the lithic material is located in two areas, next to the elephant skull and vertebras (Fig 10).

The concentration of lithic pieces in those small areas is important. For example, in the skull area (about 1,5 m2 x 15 cm thick), 236 pieces were found, 88 during the excavation and 148 by sieving. The number of pieces located in the vertebras area was less than that of the skull area. The presence of five refittings in the excavated area indicates that the material had not suffered any significant perturbation. This is supported by the results of use wear analysis and by sieving sediment. We set up a protocol, saving all the sediment with a 0,1 mm sieve. This enabled us to collect 274 implements. Among these very small pieces we found some small flakes, which likely came from retouch or knapping activities at the site.

### Remarks on the lithic assemblage

The material of Ficoncella, even though it’s still very incomplete, provides some extremely interesting information. The technological features are both very original and well integrated in the Lower Palaeolithic variability. The absence of a handaxe is not so surprising given the technical trends known in Italy and Southern Europe for this time period. The presence of small tools industry is consistent with findings in western Europe (Isernia [94]; Quarto delle Cinfonare [95], Caune de l’Arago [96]), central Europe (Vertesszölös: [97–98]; Bilzingsleben: [99–98]), and Levant (Quesem Cave: [100]; Revadim: [13])) during Lower Palaeolithic.

Good preservation of the site and a detailed excavation protocol enable us to reach different types of data and observations. The functional data show a great range of different possible activities. The technical abilities, evidenced by the precision in core preparation, flaking and retouch, are remarkable. This may help to renew our perception of the lithic industries without handaxes; something that is too often ignored. However, the lack of a reduction sequence hinders our understanding of the entire technical system.

## Discussion

### Timing of the Ficoncella site formation within the framework of the Quaternary sea-level changes

Based on the stratigraphic and tephrochronologic results, it is possible to reliably outline the time range within which the sedimentary succession of the Ficoncella site developed and its relationship to Quaternary sea-level oscillations. In the investigated succession, two major erosional surfaces have been recognized. The older surface separates the continental middle Pleistocene deposits from the marine lower Pliocene deposits. This surface coincides with the base of the incised valley and constitutes the expression of a sequence boundary (terminology from sequence stratigraphy [101–102]; it represents a diachronous basal valley-fill surface that records a long period where alternating phases of rising and falling base level are strictly related to the Neogene relative sea-level changes. Taking into account our tephrochronological results and literature data, the FIC 1 unit is considered to be deposited during the interval time between the MIS 14 and 13, a period of characterized by eustatic sea-level rise. This is in good agreement with the climatostratigraphic position of the tephra FIC-12.90, which is correlated to a position at ~ 490 ka within the Acerno pollen record that slightly precedes the peak of *Quercus* pollen at the culmination of MIS 13.1 (Fig 5). The FIC 1 unit probably records the older portion of the Synthem 1 by De Rita et al. [55]. Considering the sequence stratigraphic scheme for the Latium Tyrrhenian margin proposed by Milli [54], and more recently by Milli and Palombo [103] and Milli et al. [83], the FIC 1 unit likely represents the product of sedimentation during the final phase of the transgressive PG4 sequence (Ponte Galeria 4).

The second erosional surface is located at the base of the FIC 2 unit. It has the same significance of the previous one in that it constitutes a diachronous basal valley-fill surface that represents a sequence boundary. Based on new age of this unit together with the overlying FIC 3 unit, we suggest that FIC2 and FIC3 were deposited during the interval time between the MIS 13–12, corresponding to a period of eustatic sea-level fall. Both of these units are part of the Synthem 1 by De Rita et al. [55], and belong to the PG5 sequence (Ponte Galeria 5) by Milli and Palombo [103] and Milli et al. [83].

All data considered, the unit FIC 1 and its faunal and archaeological remains can be dated within the narrow temporal windows between ~500 ka, i.e., the age of the tephra FIC-12.90, and ~490 ka, i.e., the age of the MIS 13.1 which precedes the sea-level fall of the MIS 13-MIS 12 glacial period.

### Human and other agents in the site history

Continued research helps improve our knowledge of the oldest human settlements in Europe. At present, several sites that preserve traces of early human groups in the Lower and Middle Pleistocene have been found in various regions of Europe. They are often open-air sites with the presence of faunal remains associated with lithic industries, but there can be very different degrees of conservation, dynamics of site formation, environment and climate, faunal associations and technical systems materialized in lithic industries.

We will now discuss the question of site formation dynamics. It is necessary to assess the taphonomical process to be able to propose hypotheses regarding the modalities of occupation. Is the lithic assemblage *in situ*? What is the relationship between the human occupation and the fauna accumulation? How can we appraise the role of carnivore, whose frequentation is attested by the presence of a coprolite (probably hyena), and gnawing marks on some elephant bones? Our interest is to evaluate the accumulation temporalities of the different agents that constitute the site (human, fauna, carnivore, sediments, etc.).

Some information comes directly from the lithic assemblage itself [104–105]. In the case of Ficoncella, the spatial analysis shows that the lithic artefacts were located in two small areas (concentrated near the skull and vertebras of the elephant). The consistency of the reduction sequence, the presence of refitting and the identification of 21 raw material units (obviously human knapped and not local) testify that the lithic assemblage was not subject to important post depositional phenomena. Indeed, this hypothesis is reinforced by the presence of very small chips (retouch and other technical flakes) that may indicate that knapping took place in situ. The preliminary analysis on the orientation and the inclination of the lithic implements shows random distribution that does not indicate a clear preferential orientation of the pieces on the excavation [93]. All this data are coherent with the use wear analysis that observes good preservation of material that suffered only slight soil abrasion, as evidenced by a light sheen on the surface of many tools. On the other hand we observe the absence of a large element in the assemblage, with the exception of the limestone flake. We can’t exclude that part of the assemblage is missing for a post depositional reason, such as a gravitational event for example. Even if the dimension of the selected pebble in quartz and flint is also small. If we need to be careful because of the reduced dimension of the excavation area, the current data recovered on the lithic assemblage is interpreted to suggest that lithic accumulation was a short event without massive perturbation.

The sedimentological data indicate that the floodplain environment (Fic1) was affected by flood events alternating with subaerial exposure and drying. The interpretation of the lithic accumulation is coherent with this setting: human occupation likely took place during one of the dry, exposure events, and may have been buried relatively quickly during a flood event. It is difficult to assert that the human occupation is strictly coeval with the fauna accumulation. The *Palaeoloxodon* carcass is in anatomical connection, at least for the upper part (skull and tusk). But as known, the taphonomical process of the elephant carcass is complex [106–103–107]. A study of modern African elephants shows that during the scavenging period, several carnivores can follow each other on the carcass for almost a year [108]. The coexistence of humans and carnivores, especially hyaenas (and may be their competition for consumption of herbivore carcasses), is reported from a number of Pleistocene sites in Eurasia [14], although the sequence of humans vs carnivores exploitations is often difficult to ascertain. In this context, it is not possible to constrain the role of humans in this process, as the temporality of these two events was probably different. The study of the *Palaeoloxodon* remains shows no evidence of cut marks, nevertheless we cant exclude a human intervention on the carcass. In fact, direct evidence of butchery practices generated by humans on elephants is sometimes difficult to identify and ascertain because of the thickness of the skin and muscular masses [11–17–16–20]. However, given that the localization of the artefacts is very close to the carcass and in the sediment during burial, it seems likely that this occupation was in some extent linked to the *Palaeoloxodon* carcass. Indeed, the relationship between human and elephant during Lower Palaeolithic is well recognized in literature [21–109–5–11–12–110]. Especially in Southern Europe and Italy, thought often the association could be accidental, while an actual exploitation of elephant carcasses by human groups it sometimes difficult to ascertain. In Italy, scavenging-butchering practices on elephants have been reported at a few sites, sometimes inferred from the spatial distribution of lithic implements with respect to elephant remains (Notarchirico [18], La Polledrara di Cecanibbio [21], Bucine [111–112]), and rarely because of the presence of cut and/or percussion marks (Castel di Guido [113]).

In summary, we propose to interpret the Ficoncella site as the result of different frequentation in a short time range in a floodplain environment. Between the death of the elephant and its burial, the area was probably frequented alternatively in a very short period of time by carnivorous animals and humans, the latter of which prepared and used lithic tools.

## Concluding Remarks

The new results enable us to constrain the age and environmental context of Ficoncella site. The ^40^Ar/^39^Ar dating, tephrochronologic and geologic data concur to attribute the archaeological level to a highstand phase of MIS 13 or ca. 500 ka. Indeed, the unit FIC 1 and the related faunal and archaeological remains can be dated within the narrow temporal windows between ~500 ka, i.e., the age of the tephra FIC-12.90, and ~490 ka, i.e., the age of the MIS 13.1 which precedes the sea-level fall of the MIS 13-MIS 12 glacial period. In the light of the taphonomical data, we propose that the mammal remains and lithic components were buried shortly after emplacement by alluvial/floodplain deposits. Even if we consider that the human occupation was linked to the elephant carcass (scavenging?), it is impossible to know the order of their frequentation [107] and their degrees of competition [14]. The quick burial of the lithic implement, evidenced by the sedimentological data, as well as by the spatial distribution of the material, suggests that the human occupation may be considered a snapshot. Brief occupations are very rare during the Lower Palaeolithic. To be able to characterize more precisely the occupation dynamics at Ficoncella, additional data from new and more extensive archaeological excavation are needed.
